# Pharmacokinetic evaluation of Chalcone derivatives with antimalarial activity in New Zealand White Rabbits

**DOI:** 10.1186/s13104-021-05684-8

**Published:** 2021-07-08

**Authors:** Shweta Sinha, Ajay Prakash, Bikash Medhi, Alka Sehgal, Daniela I. Batovska, Rakesh Sehgal

**Affiliations:** 1 Department of Medical Parasitology, Post Graduate Institute of Medical Education and Research, Chandigarh, 160012 India; 2 Department of Pharmacology, Post Graduate Institute of Medical Education and Research, Chandigarh, India; 3 Department of Obstetrics & Gynecology, Government Medical College & Hospital Sector 32, Chandigarh, India; 4 Institute of Organic Chemistry With Centre of Phytochemistry, Bulgarian Academy of Sciences, Sofia, Bulgaria

**Keywords:** Malaria, Chalcones, RP-HPLC, Bioavailability

## Abstract

**Objective:**

Malaria is a major global health concern with the urgent need for new treatment alternatives due to the alarming increase of drug-resistant *Plasmodium* strains. Chalcones and its derivatives are important pharmacophores showing antimalarial activity. Determination of the pharmacokinetic variables at the preliminary step of drug development for any drug candidates is an essential component of in vivo antimalarial efficacy tests. Substandard pharmacokinetic variables are often responsible for insufficient therapeutic effect. Therefore, three chalcone derivatives, 1, 2, and 3, having antimalarial potency were studied further for potential therapeutic efficacy.

**Results:**

In vivo pharmacokinetic studies of these three derivatives were performed on New Zealand White rabbits. The three derivatives were administered intra-peritoneally or orally at effective dose concentration and blood samples at different time points were collected. The determination of drug concentration was done through reverse phase-high performance liquid chromatography. The peak plasma concentration of derivative 1, 2, and 3 were 1.96 ± 0.46 µg/mL (intraperitoneal route), 69.89 ± 5.49 µg/mL (oral route), and 3.74 ± 1.64 µg/mL (oral route). The results indicate a very low bioavailability of these derivatives. The present study gives a benchmark to advance the investigation of more derivatives in order to revamp the pharmacokinetic variables while maintaining both potency and metabolic constancy.

**Supplementary Information:**

The online version contains supplementary material available at 10.1186/s13104-021-05684-8.

## Introduction

Malaria caused due to *P. falciparum*, the most virulent species, is a remarkable reason for the morbidity and mortality all over the globe [1]. Artemisinin-combination therapy is the most recommended therapy till now for *P. falciparum* malaria. However, the appearance of artemisinin resistant strains of *P. falciparum* in the various part of the greater Mekong subregion has raised major concern because of the lack of appropriate alternative therapies [2]. This shows the necessity for the development of a unique generation of curatives that can target the threats of the malaria elimination agenda [3]. Malaria chemotherapy has a strong historical link to plant and natural products, in this context chalcones an important pharmacophore has been explored extensively from past decades for antimalarial activity [4].

Chalcones, or 1,3-diaryl-2-propen-1-ones, belongs to the flavonoid family and chemically they consist of open-chain flavonoids in which the two aromatic rings are joined by a three-carbon a, b-unsaturated carbonyl system. The radical dousing ability due to the presence of the phenolic groups in numerous chalcones has heightened the attentiveness in the utilization of these compounds or their derivatives in the form of food preservatives or drugs [5]. Primeval therapeutic application of chalcones is known to be linked with the utilization of several-year-old herbs and plants for the remedy of distinct medical ailments [6]. Chalcones, either in form of natural or synthetic, are well known to illustrate an extensive spectrum of biological activities, including antimalarial [7], anti-inflammatory [8], cytotoxic [9, 10], anticancer properties [11, 12], modification of P-glycoprotein-mediated multidrug resistance [13], and antioxidant [14]. Due to simple chemical structure, accessibility, and numerous methods of cyclization, this specific class of compounds has meant as central in the quest for lead molecules having therapeutic implication. With the evident of therapeutic application, our groups have synthesized a series of new derivatives of chalcones which were screened for antimalarial activity in vitro culture system [15, 16]. However, because of poor pharmacokinetic variables such as short half-life, low bioavailability, fast metabolism, and accelerated clearance ensuing in a short period of action, restricts the drug from being deployed for the actual therapeutic effect [17]. Additionally, in the vast majority of the cases, antimicrobial therapeutics which exhibit immense antimicrobial activity under in vitro, fails to reveal their therapeutic activity in the experimental infection models [18]. For this cause, it is critical to appraise the pharmacokinetic variables of potential lead compounds at the preliminary stage of drug development [17]. Considering these facts, the three potent chalcone derivatives having antimalarial activity were studied further for pharmacokinetic evaluation.

## Main text

### Materials and methods

#### Chemical and reagents

**Fig. 1:**
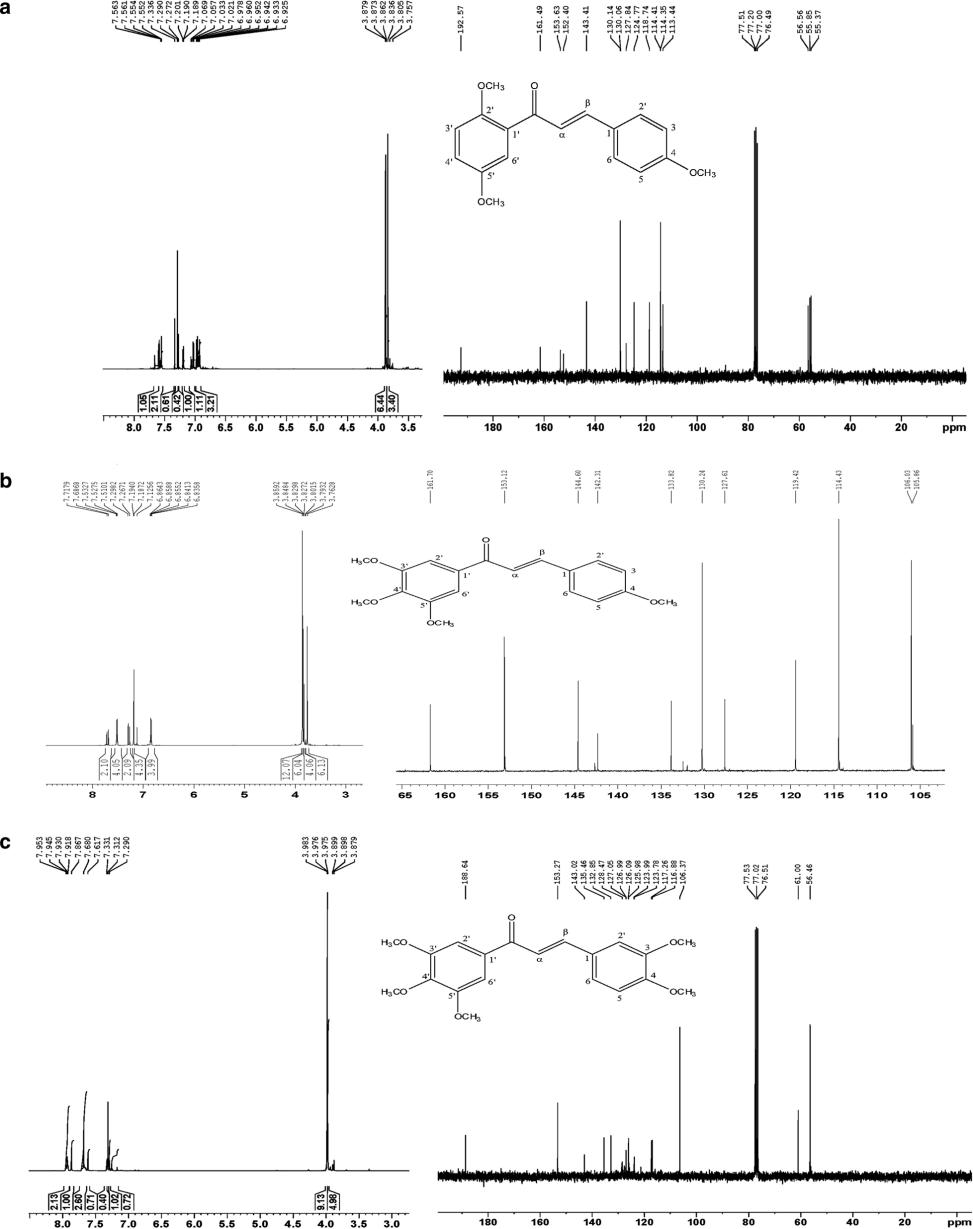
NMR Spectrum and molecular structure of chalcone derivatives. **a** NMR spectrum (600 MHz, CDCl_3_) and molecular structure of compound 1, **b** NMR spectrum (500 MHz, CDCl_3_) and molecular structure of compound 2, and **c** NMR spectrum (600 MHz, CDCl_3_) and molecular structure of compound 3

Pure form of Chalcone was bought from Sigma Aldrich and was utilized as in same form considering as a reference standard. The methanol and water used in the experiments were of HPLC analytical grade. The three chalcone derivatives namely,

(E)-1-(2,5-Dimethoxyphenyl)-3-(4-methoxyphenyl)prop-2-en-1-one, (1);

(E)-(3,4,5-Trimethoxyphenyl)-3-(4-methoxyphenyl)prop-2-en-1-one, (2); and.

(E)-1-(3,4,5-Trimethoxyphenyl)-3-(3,4-dimethoxyphenyl)prop-2-en-1-one,(3),were synthesized as delineated formerly by our group [15] and NMR characterization of these derivatives with their molecular structure are shown in Fig. 1a–c and detailed outline of NMR spectral reading is given in Additional file 1.

#### Pharmacokinetic studies

The ten healthy adult New Zealand White rabbits weighing between 1.75–2.75 kg of either sex were procured from the institute Advanced Facility for Small Animal Research, PGIMER Chandigarh, after approval from the Institutional Animal Ethics Committee Ref. No. 69/IAEC/418 as per the Committee for the Purpose of Control and Supervision of Experiments on Animals (CPCSEA) guidelines. The animals were housed in polypropylene cages under standard laboratory conditions at controlled temperature (25 ± °C) and 12-h light/dark cycles until the end of the experimental period. Animals were given free access to diet and water in a room and were fasten for at least 12 h prior to experiment. To achieve meaningful statistical results, we used a minimally sufficient number of animals in all cases. A fifteen-days washout period was carried out, which allows animals to completely recover and clear off the administered derivatives.

A total of ten healthy adult New Zealand White rabbits were divided into three groups; (i) derivative 1 treated group, (ii) derivative 2 treated group, and (iii) derivative 3 treated group. The whole experiment was performed in two batches with each batches containing n = 3 animals per group. The second batch with same groups were repeated after giving fifteen-days washout period which helps animals to recover and clear off previous administered derivatives. The three chalcone derivatives were administered at effective dose concentration [dose extrapolated from in vitro study, [15] as per OECD GLP compliance. A single dose of each chalcone derivatives 1, 2 & 3 (suspended in 0.5% carboxymethyl cellulose) were administered to each animal in the early morning at 7:00 am, which was noted as 0 h and then blood samples (1 mL) of animals were collected at specific time intervals of 0, 0.25 h, 0.5 h, 1 h, 2 h, 4 h, 6 h, 8 h, 12 h, 24 h, 36 h, 48 h from marginal ear vein in heparin containing vials. These derivatives were either administered orally through orogastric tube or intraperitoneally. All blood samples were collected from the marginal ear vein after topical anaesthesia with 4% lignocaine solution. No euthanasia procedure was required during the study, and after completion of experiments all animals were kept in rehabilitation centre according to CPCSEA guidelines, in the institute Advanced Facility for Small Animal Research, PGIMER, Chandigarh. Plasma samples for the determination of drug concentration were prepared for RP-HPLC analysis.

#### Assay procedure

##### Preparation of Plasma Sample

Rabbit blood samples were accumulated in heparin contained dry evacuated tubes from marginal ear veins of healthy rabbits as per the standard operating procedure. These samples were centrifuged within 1 h of collection, for segregation of plasma at 1500 rpm for 10 min. The segregated plasma was preserved at 20 °C till assay. Prior experimental analysis the deproteinization of the plasma samples was done in the mixture of methanol and water (90:10 v/v), then vortexed for at least 10–15 min, after while centrifuged for at least 15 min at 6000 rpm, and finally supernatants were pipette-out in a new vial. The supernatants were then spiked with the measured volume of diluted stock solutions of chalcone making up to final concentrations of 10–100 μg/mL. Each sample of 20 μL volume was injected through a Rheodyne injector in the instrument and the effluent was observed at 310 nm.

##### Standard preparation

A concentrated (100 μg/mL) stock solution was obtained by solubilizing 10 mg of chalcone in 10 mL of methanol and then the final volume was added up to 100 mL with the mobile phase in 100 mL volumetric flask. The working solution of chalcone was prepared by diluting the stock solution with the mobile phase. The same procedure was followed for plotting the calibration curve for all the three chalcone derivatives, (Additional file 2: Figure S1).

##### Sample preparation

Each sample has been collected from rabbits at a different time interval and plasma was separated and either processed for assay procedure or stored at – 20 °C in deep freezer till experiment. On the day of the experiment, 100 µL of plasma from each vial was transferred to another and mixed with 100 µL of acetonitrile (HPLC analytical Grade). Then was kept for 5 min at room temperature and then centrifuged at 7000 rpm for 10 min. Supernatant was separated and reconstituted with the mobile phase and finally sterile filter with 0.22 µm membrane filter before the assay procedure.

##### Chromatographic conditions

Chromatographic separation was achieved as mentioned earlier [19] with slight modification. Briefly, the separation was performed on RP-HPLC at ambient temperature (25 °C) by using a mobile phase consisting of methanol and water in the ratio of 90:10 (v/v) for 10 min. The mobile phase was pumped at a rate of 1.0 mL/min. The detector wavelength was set at 310 nm.

#### Pharmacokinetic variables evaluation

The experimental data and pharmacokinetic variables are shown as the mean ± standard error mean (SEM). The peak serum concentration (C_max_) and the time to achieve maximum concentration (t_max_) were retrieved from the observed concentration versus time data profile. The area under the serum concentration–time curve from time zero to the time of final measurable sample (AUC_0-48_) was calculated using the linear trapezoidal method [20].

### Results and discussion

**Fig. 2:**
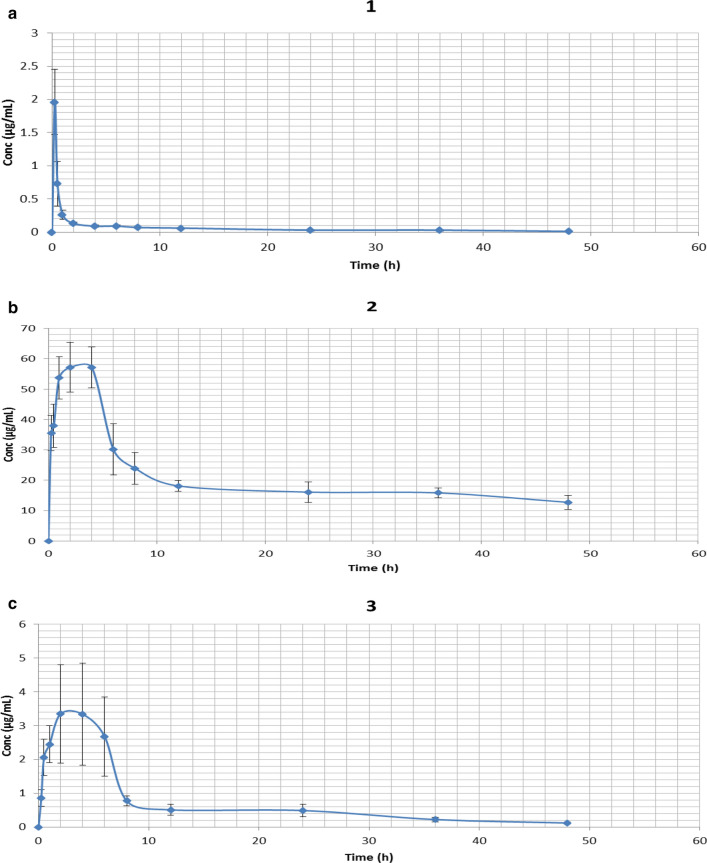
Plasma concentration–time profile for chalcones derivatives 1, 2 and 3. **a** A single 3.64 mg/kg intraperitoneal dose administration of derivative 1 in the rabbit (n = 6). **b** A single 4.85 mg/kg oral dose administration of derivative 2 in the Rabbit (n = 5). **c** A single 3.64 mg/kg oral dose administration of derivative 3 in the rabbit (n = 6). Data represented as (mean ± SEM)

One of the critical steps in the assessment of therapeutic candidates is a relative investigation of pharmacokinetic variables. The timing of compound administration and metabolic stimulation adds to the result of viability testing in vivo [21, 22]. Decreased absolute bioavailability, low distribution, accelerated metabolism as well as elimination, of chalcones, are the primary issues in planning new therapeutics based on their structure. In this study, the peak plasma concentration of derivative 1 was 1.96 ± 0.46 µg/mL achieved at 0.33 ± 0.05 h succeeding a single 3.84 mg/kg intraperitoneal dose in (Fig. 2a). The mean CL and V_d_, of derivatives **1** were 0.28 ± 0.08 L/h and 7.31 ± 0.29 L, respectively. However, the peak plasma concentration of **2** was 69.89 ± 5.49 µg/mL achieved at 3.4 ± 0.79 h following a single 4.85 mg/ kg oral dose, (Fig. 2b) and **3** was 3.74 ± 1.64 µg/mL achieved at 2.83 ± 0.87 h following a single 3.64 mg/ kg- oral dose, (Fig. 2c). The mean CL and V_d_, of derivative **2** were 0.15 ± 0.05 L/h and 10.72 ± 0.66 L, and for **3** was 0.53 ± 0.10 L/h and 9.12 ± 0.73 L respectively. Various pharmacokinetic parameters for chalcone derivatives 1, 2, and 3 are summarized in (Table 1).

**Table 1:** Pharmacokinetic parameters (mean ± SEM) after single intraperitoneal/oral dose of three (1, 2 & 3) chalcone derivatives

Pharmacokinetic Parameters	Derivative 1 (mean ± SEM)	Derivative 2 (mean ± SEM)	Derivative 3 (mean ± SEM)
Administered Dose (mg/1.5 kg Rabbits)	5.46	7.28	5.46
Administration Route	Intraperitoneal	Oral	Oral
C_0_ (µg/mL)	0.966 ± 0.025	0.986 ± 0.004	0.944 ± 0.007
C_max_ (µg/mL)	1.96 ± 0.46	69.89 ± 5.49	3.74 ± 1.64
t_max_ (h)	0.33 ± 0.05	3.4 ± 0.79	2.83 ± 0.87
t_1/2_ (h)	12.60 ± 2.09	93.32 ± 32.59	13.30 ± 1.67
AUC_0–48_ (µg.h/mL)	2.94 ± 0.83	3755.16 ± 738.71	14.16 ± 3.78
K_e_	− 0.035 ± 0.026	− 0.013 ± 0.004	− 0.056 ± 0.007
CL (mL/min)	0.28 ± 0.08	0.15 ± 0.05	0.53 ± 0.10
V_d_ (L)	7.31 ± 0.29	10.72 ± 0.66	9.12 ± 0.73

*C*_*0*_ Concentration of administrated drug at time zero, *C*_*max*_ Peak plasma concentration, *t*_*max*_ time to reach the peak plasma concentration, *t*_*1/2*_ elimination half-life, *AUC* Area under the total plasma concentration–time curve, *K*_*e*_ Elimination rate constant, *CL* Plasma clearance, *V*_*d*_ Volume of distribution

The peak concentration of derivative **2** was achieved at 3.84 h and was remain at constant concentration for 3–4 h. This may result in long half-life of **2**. This finding indicates that **2** has a longer half-life compared to other two derivatives **1** and **3** which is also similar to the attributed reason of chloroquine longer half-life where noticeable amount of the chloroquine was observed for several days in the plasma since after discontinuance of the therapy [23, 24]. Nevertheless, the drug having a longer half-life was associated to considerable tissue cohering, specifically in melanin accommodating tissues, spleen, kidney, liver, lung, and less significantly in the spinal cord and brain. Since drugs bounded to protein are not freely accessible and it cannot be easily spotted in the plasma or serum and cannot be even metabolized [25]. The observed decline in serum concentration, elimination rate constant and clearance rate of derivative 2 might be ascribed to improvement in the binding of derivative **2** to microsomal protein, which results in a lower level of unbounded drug in the serum and therefore a reduction in the above-mentioned variables. Such association gives explanation for the observed surge in the drug half-life as protein association leads to extended half-life of a drug by prolonging its liberation from the tissues [23]. Single oral administration of derivative **2** has comparatively better systemic exposure, compared to other two chalcone derivatives and also has a longer half-life. Moreover, as compared to in vitro concentration [15], compound 1 has three fold, compound 2 has tenfold and compound 3 has 25 fold increased bioavailability.

Derivative **3** has poor bioavailability. The metabolism of derivative **3** follows first order kinetics, which explains the steep fall in plasma **3** levels during the elimination phase. Moreover, after IP administration of derivative **1**, there was the rapid absorption of the derivative from the rabbit gastrointestinal tract. It was spotted in plasma from the very first blood sampling time (15 min) and quickly reached *T*_max_ (0.33 h) manifesting linear dynamics. Overall, the pharmacokinetic study indicates very low bioavailability of derivative **1** and **3** compared to **2**. This result is consistent to the previous published literature, in which the pharmacokinetic study carried out in *Sprague–Dawley* rats demonstrates exceptionally poor oral bioavailability of chalcones which could be the explanation behind its decrease antimalarial activity, to rule out parasitemia (100%) in animal models [22, 26, 27]. Moreover, drug efflux transporters of the ATP binding cassette (ABC) family of proteins display a major part in the regulation of the pharmacokinetic and pharmacodynamic properties of drugs and intriguingly, chalcones are found to be one of the effective inhibitors of P-glycoprotein (P-gp) [28], which can limit the bioavailability and ultimately their therapeutic efficacies.

### Conclusions

The data presented in this study give a benchmark to advance the investigation of more derivatives in order to revamp the pharmacokinetic variables while maintaining both potency and metabolic constancy. The study also provides a simple and reproducible RP-HPLC method for determination of chalcones in plasma which can be opted in resource limiting settings.

## Limitations

The present study focussed only on pharmacokinetic analysis of these chalcone derivatives. However, pharmacokinetic data supported by a parallel analysis of efficacy of malaria parasites in vivo would be more conclusive. Additionally, the use of rabbits for pharmacokinetic data is not as much reliable and replaceable compared to clinical pharmacokinetic studies.

## Supplementary Information

**Additional file 1. **NMR characterization of chalcone derivatives.

**Additional file 2: Figure S1.** Calibration curve for chalcone derivative 1 (a), 2 (b), and 3(c).

## Data Availability

All data generated or analysed during this study are included in this published article [and its additional files].

## Abbreviations

ABC: ATP binding cassette
AUC: Area under the total plasma concentration–time curve
C_max_: Peak plasma concentration
CL: Plasma clearance
CPCSEA: Committee for the Purpose of Control and Supervision of Experiments on Animals
IP: Intraperitoneal
NMR: Nuclear magnetic resonance
OECD GLP: Organisation for Economic Co-operation and Development, Good Laboratory Practice
RP-HPLC: Reverse phase-high performance liquid chromatography
t_max_: Time to reach the peak plasma concentration
t_1/2_: Elimination half-life
V_d_: Volume of distribution
