# Supplementary material for: Pharmacokinetic evaluation of Chalcone derivatives with antimalarial activity in New Zealand White Rabbits
Source: BMC Res Notes. 2021 Jul 8;14:264. doi: 10.1186/s13104-021-05684-8 (PMC8268181; doi:10.1186/s13104-021-05684-8)
Supplement: Supplementary file 1 — Additional file 1. NMR characterization of chalcone derivatives. [file 13104_2021_5684_MOESM1_ESM.docx]

**Additional file 1: NMR characterization of chalcone derivatives**

Compound 1: (*Е*)-1-(2,5-Dimethoxyphenyl)-3-(4-methoxyphenyl)prop-2-en-1-one: ^1^Н-NMR (600 MHz, CDCl_3_): δ 7.60 (d, 1H, H-β, *J* = 15.8 Hz), 7.52-7.56 (m, 2H, H-3, H-5), 7.27 (d, 1H, H-α, *J* = 15.8 Hz), 7.15-7.17 (m, 1H, H-6’), 7.01 (dd, 1H, *J_1_* = 3.0 Hz, *J_2_* = 9.0 Hz, H-4’), 6.91-6.95 (m, 1H, H-3’), 6.90-6.93 (m, 2H, H-2, H-6), 3.85 (s, 3H, OCH_3_), 3.84 (s, 3H, OCH_3_), 3.81 (s, 3H, OCH_3_); ^13^C-NMR (600 MHz, CDCl_3_): δ 192.6 (C=O), 161.5 (C-4), 153.6 (C-5’), 152.4 (C-2’), 143.4 (C-β), 130.1 (C-2, C-6), 127.8 (C-1’, C-1), 124.8 (C-3’, C-4’), 118.7 (C-α), 114.4 (C-3, C-5), 113.4 (C-6’), 56.6 (OCH_3_), 55.9 (OCH_3_), 55.4 (OCH_3_).

Compound 2: (*Е*)-(3,4,5-Trimethoxyphenyl)-3-(4-methoxyphenyl)prop-2-en-1-one: 1Н-NMR (500 MHz, CDCl3): δ 7.70 (d, 1H, H-β, J = 15.5 Hz), 7.51-7.53 (m, 2H, H-2, H-6), 7.28 (d, 1H, H-α, J = 15.5 Hz), 7.13 (s, 2H, H-2’, H-6’), 6.84-6.86 (m, 2H, H-3, H-5), 3.86 (s, 6H, 2xOCH3), 3.84 (s, 3H, OCH3), 3.76 (s, 3H, OCH3); ^13^C-NMR (500 MHz, CDCl_3_): δ 189.3 (C=O), 161.7 (C-4), 153.1 (C-3’, C-5’), 144.7 (C-β), 142.2 (C-4’), 133.9 (C-2, C-6), 130.3 (C-1’), 127.6 (C-1), 119.4 (C-α), 114.4 (C-3, C-5), 106.0 (C-2’, C-6’), 61.0 (OCH_3_), 56.4 (2xOCH_3_), 55.5 (OCH_3_).

Compound 3: (*Е*)-1-(3,4,5-Trimethoxyphenyl)-3-(3,4-dimethoxyphenyl)prop-2-en-1-one: ^1^H-NMR (600 MHz, CDCl_3_): δ 7.77 (d, 1H, H-β, *J* = 15.5 Hz), 7.33 (d, 1H, H-α, *J* = 15.5 Hz), 7.27 (s, 2H, H-2', H-6'), 7.26 (dd, 1H, H-6, *J_1_* = 2.0 Hz, *J_2_* = 8.3 Hz), 7.16 (d, 1H, H-2, *J* = 2.0 Hz), 6.91 (d, 1H, H-5, *J* = 8.5 Hz), 3.95 (s, 9H, 3xCH_3_O), 3.94 (s, 6H, 2xCH_3_O); ^13^C-NMR (600 MHz, CDCl_3_): δ 189.4 (C=O), 153.1 (C-3’, C-5’), 151.4 (C-3, C-4), 149.3 (C-4’), 144.9 (C-β), 133.8 (C-1’), 127.9 (C-1), 122.9 (C-6), 119.9 (C-α), 111.2 (C-2, C-5), 110.6 (C-2’, C-6’), 60.9 (OCH_3_), 56.4 (2x OCH_3_), 56.0 (OCH_3_), 55.9 OCH_3_).
