# Supplementary figures and images for: Pharmacokinetic evaluation of Chalcone derivatives with antimalarial activity in New Zealand White Rabbits
Source: BMC Res Notes. 2021 Jul 8;14:264. doi: 10.1186/s13104-021-05684-8 (PMC8268181; doi:10.1186/s13104-021-05684-8)

## Slide 1
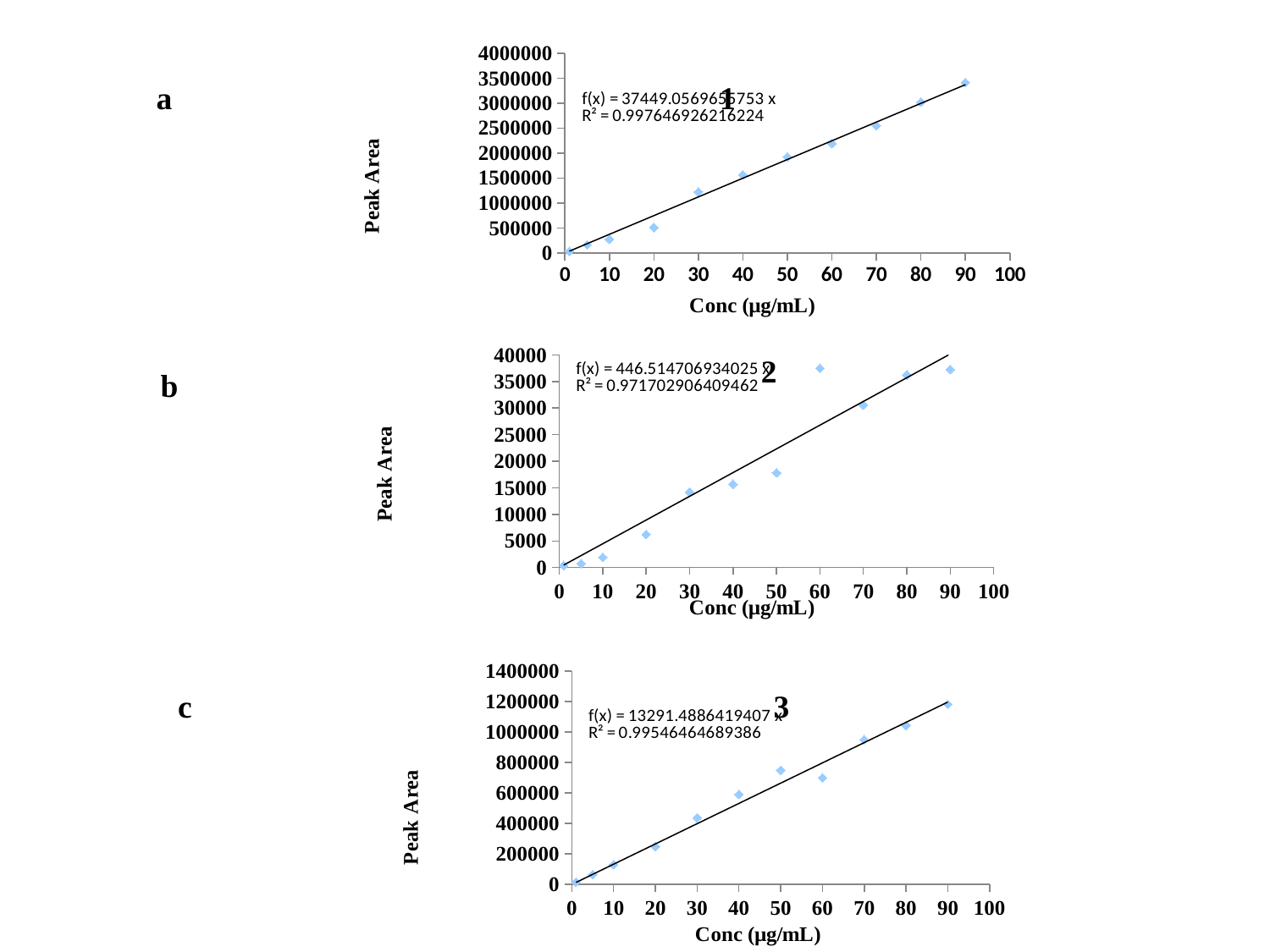

### Chart
| Category | Mean |
|---|---|a
1
### Chart
| Category | Mean |
|---|---|2
b
### Chart
| Category | Area |
|---|---|c
3

Supplement: Supplementary file 2 — Additional file 2: Figure S1. Calibration curve for chalcone derivative 1 (a), 2 (b), and 3(c). [file 13104_2021_5684_MOESM2_ESM.pptx]
